# Variant Human T-cell Lymphotropic Virus Type 1c and Adult T-cell Leukemia, Australia

**DOI:** 10.3201/eid1910.130105

**Published:** 2013-10

**Authors:** Lloyd Einsiedel, Olivier Cassar, Peter Bardy, Daniel Kearney, Antoine Gessain

**Affiliations:** Flinders University/Northern Territory Rural Clinical School, Alice Springs, Northern Territory, Australia (L. Einsiedel);; Institut Pasteur, Paris, France (O. Cassar, A. Gessain);; Centre National de la Recherche Scientifique Unité Mixte de Recherche 3569, Paris (O. Cassar, A. Gessain);; Royal Adelaide Hospital and Institute of Medical and Veterinary Science, Adelaide, South Australia, Australia (P. Bardy, D. Kearney)

**Keywords:** human T-cell lymphotropic virus type 1, HTLV-1, adult T-cell leukemia/lymphoma, ATLL, molecular epidemiology, indigenous Australians, Australia, viruses

## Abstract

Human T-cell lymphotropic virus type 1 is endemic to central Australia among Indigenous Australians. However, virologic and clinical aspects of infection remain poorly understood. No attempt has been made to control transmission to indigenous children. We report 3 fatal cases of adult T-cell leukemia/lymphoma caused by human T-cell lymphotropic virus type 1 Australo-Melanesian subtype c.

The human T-cell lymphotropic virus type 1 (HTLV-1) currently infects at least 5–10 million persons worldwide; however, this oncogenic retrovirus is not ubiquitous, and areas of high endemicity are typically separated by areas where infection is uncommon (*1*). Although 4 major molecular subtypes in specific geographic areas have been described, epidemiologic and clinical associations have been best documented for the HTLV-1a subtype, which predominates in the Caribbean region and Japan. Among HTLV-1 carriers in these regions, adult T-cell leukemia/lymphoma (ATLL) will ultimately develop in 1%–5% (*2*). Few clinical details are available with regard to infection with the Australo-Melanesian HTLV-1c subtype, which is restricted to impoverished indigenous populations in Australia and the neighboring islands of Oceania.

In Australia, HTLV-1 carriers were first reported among indigenous residents of remote desert communities in 1988 (*3*). The sole published HTLV-1 nucleotide sequence from an indigenous Australian belongs to the Australo-Melanesian HTLV-1c subtype (*4*). Genetic characterization of the few available HTLV-1c subtype strains indicates that they are relatively divergent compared with the Cosmopolitan HTLV-1a prototype. Within the *env* gene and long terminal repeat regions, for example, 7%–10% divergence has been demonstrated at the nucleotide level (*5*,*6*). Background prevalence rates among indigenous central Australian adults are thought to be from 7.2% through 13.9% (*7*,*8*). However, among those admitted to the only regional hospital, the seropositivity rate approaches 40% (*8*), and rates are even higher among patients >45 years of age (49.3% for men; 38.5% for women) (*8*). The predominant mode of transmission among indigenous Australians is thought to be through breast-feeding (*8*).

In other populations, early acquisition of HTLV-1 infection is associated with an increased risk for ATLL (*2*). High HTLV-1 prevalence rates in some indigenous Australian and Melanesian communities coupled with frequent early childhood infection with HTLV-1 should therefore be associated with a correspondingly high risk for ATLL, yet few cases of HTLV-1–associated complications have been reported from Australasia (*9*,*10*). Indeed, the clinical significance of HTLV-1 infection in Australia has been questioned, and no attempt has been made to control virus transmission among the indigenous population (*8*). We report 3 cases of ATLL in indigenous Australian patients at the Alice Springs Hospital, central Australia, in 2002 and 2010. Case-patients 1 and 3 (Aus-NR and Aus-GJ) originated from the same community, ≈450 km from case-patient 2 (Aus-GM).

## The Study

**Figure 1:**
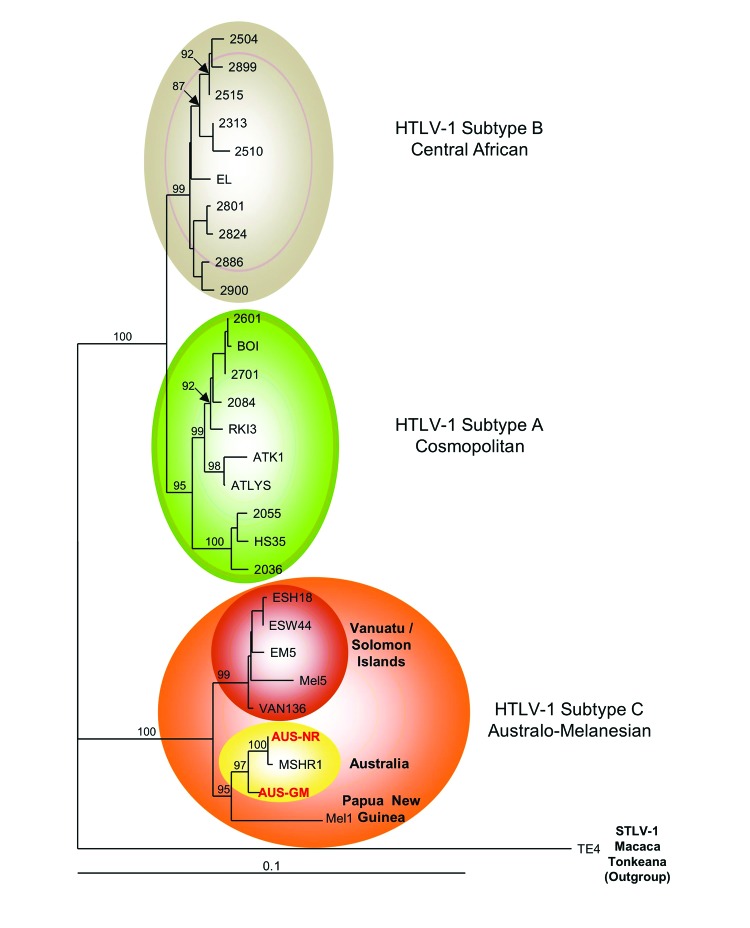
CD3 (A) and CD4 (B) cells immunostained with diaminobenzidine and horseradish peroxidase in lymph node biopsy sample from patient 2 (Aus-GM). Original magnification ×40. C) Peripheral blood cells smear from adult T-lymphocytic leukemia patient 1 (Aus-NR). Tumor cells exhibiting multilobulated nuclei (or flower cells), which are mature activated CD4+ T-lymphocytes. D) Contrast-enhanced computed tomographic image of abdomen of patient 1, revealing para-aortic lymphadenopathy. E) Contrast-enhanced computed tomographic image of patient 1, showing lytic bone lesion (L-1 veretebral body) with soft tissue involvement (arrow).

The first case-patient, a 68-year-old indigenous Australian man, reported a several-month history of limb pain, abdominal pain, and diarrhea. Peripheral blood examination revealed lymphocytosis (22.4 × 10^9^/L) with atypical lymphoid cells. The patient was seropositive for HTLV-1 by Western blot and positive for hepatitis B surface antigen. Flow cytometry of cells obtained by bone marrow and lymph node biopsies revealed a population of CD2+, CD3+, CD4+, CD5­­–, CD7−, CD8−, CD25+ cells (Figure 1, Appendix, panels A, B, wwwnc.cdc.gov/EID/article/19/10/13-0105-F1.htm).

Histopathologic appearance was consistent with a diagnosis of ATLL, and T-cell receptor rearrangement studies revealed a monoclonal band. The case was complicated by a bloodstream infection with viridans group streptococci. This patient died of liver failure, which had complicated the sepsis, 22 days after admission.

The second case-patient, a 58-year-old indigenous Australian woman, reported a 1-week history of back pain and malaise. Peripheral blood examination revealed hyperlymphocytosis (58 × 10^9^/L) caused by CD2+, CD3−, CD4+, CD5+, CD7−, and CD8− atypical lymphoid cells (Figure 1, Appendix, panel C). The patient was seropositive for HTLV-1 by Western blot. Corrected serum calcium was 3.5 mmol/L. Plain radiographs revealed lytic bone lesions, which were also apparent on computed tomography imaging of her abdomen, as was extensive lymphadenopathy and a para-aortic mass (Figure 1, Appendix, panels D, E). The results of bone marrow biopsy were consistent with ATLL, and T-cell receptor rearrangement studies revealed a monoclonal band. This patient died of sepsis 13 days after admission.

The third case-patient, a 74-year-old indigenous Australian man, reported a history of back pain; laboratory investigations revealed hypercalcemia, acute renal failure, and pancytopenia. A peripheral blood film revealed small lymphocytes with cloverleaf-shaped nuclei and prominent nucleoli. The patient was HTLV-1 seropositive by Western blot. Bone marrow biopsy revealed that the marrow was almost totally replaced by a population of CD25+ lymphoid blast cells, consistent with a diagnosis of acute ATLL. A monoclonal T-cell receptor band was observed from fresh tissue. The case was complicated by *Staphylococcus aureus* bacteremia, and the patient died of overwhelming sepsis 41 days after diagnosis.

**Figure 2:**
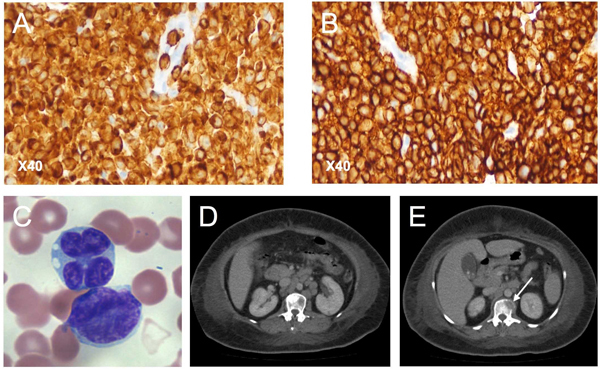
Phylogenetic tree generated on a 1,386-bp fragment of the *env* gene for 30 primate T-lymphotropic virus type 1 available sequences, including the 2 sequences generated in this work (in red **boldface**). The lymphotropic virus type 1 strains were aligned with the DAMBE program (version 4.2.13). The final alignment was submitted to the ModelTest program (version 3.6) to select, according to the Akaike Information Criterion, the best model to apply to phylogenetic analyses. The selected model was the Tamura Nei. Bootstrap values were calculated for 1,000 replicates, and the numbers at some nodes of the tree (bootstrap values) indicate frequencies of occurrence for 100 trees. The TE4 strain from *Macaca tonkeana* macaques was used as an outgroup. The branch lengths are drawn to scale, and the scale bar indicates nucleotide replacements per site. Three of the 4 major HTLV-1 subtypes are shown. Subtype c corresponds to Australo-Melanesian HTLV-1c and includes the 2 *env* sequences (Aus-NR and Aus-GM; GenBank accession nos. KF242508 and KF242509, respectively), which were obtained from patients 1 and 2, respectively. HTLV, human T-lymphotropic virus; STLV, simian T-lymphotropic virus.

Molecular characterization of the viral strain was possible for patients 1 and 2. The entire HTLV-1 *env* gene was obtained by using DNA extracted from peripheral blood buffy coats, providing a complete *env* gene sequence of the c subtype characterized from indigenous Australians. Phylogenetic analysis was performed by using a 1,386-bp fragment of *env* gene sequences (from patients Aus-NR and Aus-GM) and 28 primate T-lymphotropic virus type 1 sequences. These sequences cluster with the Australo-Melanesian c-subtype (Figure 2) and are closely related to the sole, but incomplete, HTLV-1 strain (MSHR-1) from the previously described case of an indigenous Australian (*4*). This phylogenetic study also demonstrates the existence of at least 2 subgroups strongly supported phylogenetically within the Melanesian clade: 1 comprising strains from Australia and Papua New Guinea and 1 comprising strains from Vanuatu and the Solomon Islands (Figure 2).

## Conclusions

Elsewhere, HTLV-1–associated ATLL directly causes death in as many as 1 in 20 HTLV-1 carriers (*2*). Each patient from central Australia reported here was seropositive for HTLV-1 according to stringent Western blot criteria, and the HTLV-1c variant strain was identified for both patients from which mononuclear cell DNA was available. All 3 patients died within 6 weeks of diagnosis. The patients described here had been admitted to the only regional hospital; others might have died in remote areas before transfer. Nevertheless, the estimated crude mean annual incidence of ATLL among ≈1,400 adult HTLV-1 carriers in central Australia (23.6 cases/100,000 population) is similar to that of the Caribbean region (20 cases/100,000 population) (*11*). Rates are somewhat higher in Japan (86 cases/100,000 population) (*11*); however, many indigenous Australians die during the latent period required for malignant transformation (*8*), and ATLL incidence rates in central Australia might rise as life expectancy for indigenous Australians improves.

The paucity of clinical information regarding HTLV-1–associated diseases among indigenous Australasians might reflect these persons’ limited access to medical care rather than intrinsic differences in the oncogenic potential of the HTLV-1c Australo-Melanesian subtype. Indeed, social deprivation can increase the risk for adverse outcomes among HTLV-1 carriers (*8*). For example, environmental contamination resulting from poor sanitation increases risk for co-infection with *Strongyloides stercoralis* roundworms, which shortens the latent period for malignant transformation (*12*). Whether co-infection with *S. stercoralis* parasites contributed to the development of ATLL in the patients reported here is uncertain. However, *S. stercoralis* parasites are endemic to central Australia (*8*), and anthelminthic prophylaxis has been suggested as a way to reduce risk for ATLL among HTLV-1 carriers residing in other *S. stercoralis* parasite–endemic areas (*13*). Social deprivation might also increase the risk for HTLV-1–associated pulmonary disease. In residents of developed countries, this disease is typically subclinical; however, in indigenous Australians, it appears as multifocal bronchiectasis, resulting in substantial illness and death at a median of 44.5 years of age (*14*).

The occurrence of 3 cases of ATLL in indigenous central Australian adults, together with previous studies that have demonstrated high HTLV-1 seropositivity rates (*8*), endemic strongyloidiasis (*8*), and high prevalence of HTLV-1–associated bronchiectasis (*14*), confirm that HTLV-1–associated diseases contribute to illness and death among indigenous Australians. These findings demand a public health response to control HTLV-1 transmission, particularly to indigenous children who are at greatest risk.
